# Subjective Wellbeing, Work Performance and Lived Experience of Zanzibari Women Entrepreneurs with Uncorrected Functional Presbyopia: A Pre-Post Mixed-Methods Study

**DOI:** 10.1080/09286586.2023.2279102

**Published:** 2023-11-07

**Authors:** Ving Fai Chan, Michelle Fernandes Martins, Omar Juma Othman, Ai Chee Yong, Damaris Mulewa, Christine Graham, Carlos Price-Sanchez, Ronnie Graham, Adrianna Farmer, Eden Mashayo, Fatma Omar

**Affiliations:** a Centre for Public Health, School of Medicine, Dentistry and Biomedical Sciences, Queen’s University Belfast, Northern, Ireland, UK; b Programmes Department, Vision Aid Overseas, London, UK; c College of Health Sciences, University of KwaZulu Natal, Durban, South Africa; d Department of Primary Eye Care Unit, Ministry of Health, Zanzibar, Tanzania; e Independent researcher, Nairobi, Kenya; f Research Department, Vision Care Foundation, Dar-es-Salaam, Tanzania

**Keywords:** Lived experience, presbyopia, subjective wellbeing, women entrepreneurs, work performance

## Abstract

**Purpose:**

Uncorrected presbyopia has been shown to reduce Zanzibari women’s quality of life. In this mixed-methods study, we examined the subjective wellbeing and self-reported work performance among older women entrepreneurs with functional presbyopia before and shortly after correction, and how poor vision at close distance affected their daily lives.

**Methods:**

Women entrepreneurs underwent eye examination to identify those with uncorrected functional presbyopia. Their subjective wellbeing and work performance were both measured in Cantril’s ladder. Ready-made glasses were then provided and 30 minutes to an hour later, their subjective wellbeing and work performance was remeasured. Twenty women entrepreneurs were interviewed to understand their lived experience with uncorrected presbyopia.

**Results:**

Two-hundred-seventeen women entrepreneurs were included in the survey (mean age 51.6 years, SD 8.64). Women entrepreneurs had a mean subjective wellbeing score of 3.32 (SD 1.10) pre-correction and 5.99 (SD 1.13) post-correction (*p* < .001), and a mean self-rated current work performance score of 4.62 (SD 1.36) before correction and 5.47 (SD 1.35) post-correction (*p* < .001). One-hundred-and-ninety (87.6%) and 121 women entrepreneurs (55.8%) rated their current subjective wellbeing and work performance scores < 5, respectively. Around 1/4 of women entrepreneurs reported having severe difficulties with near tasks.

**Conclusion:**

Poor vision at a close distance caused by uncorrected functional presbyopia negatively affected economic, physical and psychosocial aspects of women entrepreneurs’ lives. Subjective wellbeing and self-reported work productivity scores improved significantly shortly after presbyopia was corrected. More research with longer follow-up is needed to understand the full benefits of correcting presbyopia.

## Introduction

Presbyopia is a common age-related eye condition among adults 40 years and older, wherein the eye’s crystalline lens loses its elasticity, reducing the clarity and focus of near vision.^1^ Uncorrected presbyopia is estimated to affect 510 million people worldwide,^2^ with low- and middle-income countries (LMICs) disproportionately impacted by the disease.^3^ Significant inequality has been found between genders with 45% of men having uncorrected presbyopia versus 55% of women.^4^ Furthermore, women also develop presbyopia earlier than men on average.^5^

Studies conducted in rural China,^6^ Tanzania,^7^ and Nigeria^8^ show that presbyopia increases the difficulty of daily activities and reduces an individuals’ quality of life (QoL). Other studies conducted in LMICs found that uncorrected presbyopia negatively affected work performance and reduced income.^9,10^ Conversely, correcting presbyopia was shown to improve work productivity and increase income.^10,11^

In Zanzibar, the prevalence of presbyopia amongst people 40 years and older is as high as 89.2%.^12^ Despite this, correction rates remain low (17.6%) due to common barriers such as insufficient patient funds and the maintenance of eye health being considered low priority.^12^ Women with presbyopia in Zanzibar and those who were illiterate reported great difficulty performing near tasks and had low QoL scores.^12^ Correcting presbyopia with glasses improved individuals’ QoL significantly, with effect sizes of 3.9.^12^ Similar positive findings were observed among South African textile workers.^13^ However, there is still insufficient research on the effects of its correction on the QoL, especially in LMICs.^14^ Our study focused on older women entrepreneurs (WEs) in Zanzibar who face several inequalities.

Compared to men, Zanzibari women support twice as many unemployed persons per household, are twice as likely to be uneducated, three times as likely to be unemployed and are paid 30% less working in the same jobs.^15,16^ Only 16% of women own bank accounts, and a mere 9% own land and assets, often controlled by male family members.^17^ In this study, we studied the impact of uncorrected presbyopia to focus on the WEs’ subjective wellbeing (SWB) using a mixed-method approach to gain a more nuanced understanding of the lived experience of WEs with uncorrected presbyopia, many of which rely on income from craft-making which require good near vision.

SWB is used to understand “how people experience and evaluate their lives and specific domains and activities in their lives”.^18^ This indicator is widely used to monitor populations’ economic, social, and health conditions as well as to inform policy decisions across these domains.^19^ However, our literature review showed that no similar study had been conducted on eye health. This mixed-methods study will allow us to i) examine the SWB among older WEs with functional presbyopia before and shortly after correction and ii) examine their self-reported work performance before and shortly after their correction and iii) have a nuanced understanding how poor vision at a close distance affected their daily lives, which quantitative analysis might miss.

We hypothesised that: a) uncorrected presbyopia posed a significant challenge to WEs’ lives and work, resulting in low SWB scores before correction (score <5); b) WEs with uncorrected presbyopia would report low work performance (score <5); and c) WEs would perceive a significant improvement in SWB and work performance scores shortly after presbyopia correction.

## Material and methods

This study received approvals from the Ethics Committees from the Zanzibar Human Research Institute (ZAHREC/04/PR/MARCH/2022/12), Zanzibar Office of Government Chief Statistician (6221C2601263D) and Queen’s University Belfast (MHLS 22_72). Guidelines of Declaration of Helsinki were followed. We obtained the participants’ written informed consent before the interviews were conducted.

### Quantitative study component

WEs are woman business owners registered at the cooperatives in Zanzibar. They produce merchandises from tailoring and sewing, weaving, pottery, farming, and making soaps and generate income through selling their merchandises. The participant inclusion criteria were: a) WEs aged 35 years and older; b) with uncorrected presbyopia; and c) presbyopia that could be corrected with a pair of ready-made spectacles. Study participants were recruited using finite sampling through an eyecare service delivery programme provided to WEs through local cooperatives. All WEs 35 years and older (*N* = 313) from the registered cooperatives were invited to attend a vision screening to determine their eligibility. Initially, 278 women attended the eye examination. Sixty-one women were excluded because they either did not have presbyopia, were younger than 35 years old, or had other ocular morbidities (cataracts, suspected glaucoma and retinal disorders). Therefore, a total of 217 WEs participated in the quantitative study.

The data collectors first administered survey questionnaires to the WEs to collect demographic information. Each participant’s presenting distance vision was then screened one eye at a time using a modified Snellen Tumbling E-Chart. None of them were current spectacle wearers. Those who failed distance vision screening (could not identify at least four out of five letters on the 6/12 line) were further examined using a direct ophthalmoscope to determine the cause of vision impairment. If spectacle correction was required, subjective refraction was performed to determine each participant’s prescription. Those who passed distance vision screening had their near vision tested at their usual working distance. Individuals with vision impairment due to uncorrected refractive error that was correctable to better than 6/12 were also tested at their usual working distance. The WEs were considered to have failed their near vision screening if they could not read N8 at their usual working distance. These WE’s were then tested further to determine if their presbyopia could be corrected with near spectacle correction. Uncorrected presbyopia was defined as presenting near vision which failed N8 at usual working distance but was correctable to better than N8 with near spectacle correction. An ophthalmic clinical officer, and a public health optometrist supervised all screening and examination procedures.

Women were then asked to rate their SWB and work performance using a 10-point Cantril’s ladder (10 represented the best possible wellbeing/work performance; 1 represented the worst possible wellbeing/work performance). Additionally, WEs were asked to rate how much presbyopia affected their daily lives on a 5-point Likert scale (1 represented none or never; 5 represented all the time). Categorisation of baseline responses to questions on the effects of presbyopia on the WEs’ daily lives was regrouped *a posteriori* into three categories (1= None or mild difficulty/Never or rarely/Definitely or mostly true/None or a little of the time; 2= Moderate Difficulty/Sometimes/Not Sure/Some of the time; and 3= Severe difficulty or cannot do it at all/Often or very often/Mostly or definitely false/Most or all of the time) since cell counts were predominantly < 5. Subsequently, ready-made near spectacles were prescribed. The women were then asked to rate their SWB and work performance shortly (30 minutes to an hour) after their eyesight was corrected. All quantitative data analysis was carried out by global eye health specialists in the Statistical Package for the Social Sciences version 24 (SPSS-24). The mean SWB and work performance scores for WEs before and immediately after vision correction were calculated. The mean difference between the two SWB and work performance scores was calculated, respectively, with a paired sample t-test to assess the immediate change in wellbeing when eyesight was corrected. The proportions of WEs who reported Cantril’s scores of less than 5 for wellbeing and work performance were determined.

Multinomial regressions were conducted to assess factors associated with poor scores. The significance was tested with chi-square tests, with a significance level set at 5%.

### Qualitative study component

Twenty-four WEs were identified for the semi-structured interviews using quota and heterogeneity sampling. These women were selected based on their demographics: region of habitation, age range, and craft medium. Eight participants were included from each of the three crafts (beading/weaving, pottery and tailoring) to ensure a diverse range of responses. Provided the sample criteria allows one to reflect on the transferability of this study to other contexts.^20^ Participants between the ages of 35 and 39 were excluded from semi-structured interviews since preliminary data revealed that these women did not have ‘functional’ presbyopia (they were able to perform craftwork at normal working distances).

Two interviewers conducted and recorded the semi-structured interviews in Swahili to ensure accuracy. Audio recordings were transcribed in Swahili, translated into English and then back-translated by a public health specialist and a Swahili-speaking analyst. The interviews explored how vision affected their work by asking, “Before you were provided with near glasses, did you have any problems doing your chores at work?”. Probing questions were used to understand how poor vision affected different aspects of their daily lives.

Interview data were analysed using inductive thematic analysis.^21^ Relevant information from the English translation of interview transcripts was manually extracted onto Microsoft Excel® Spreadsheets. The two analysts first read the transcripts and familiarised themselves with the data.^22^ They identified keywords and developed preliminary condensed meaning units, sub-themes and themes from the first five transcripts. Any disagreements were then discussed with the chief investigator. The process was repeated for the rest of the transcripts. After both analysts had completed the process, a final list of sub-themes, themes, and megathemes were determined.

## Results

### Findings from survey questionnaires

#### Demographic profiles of women entrepreneurs who participated in the survey

A total of 217 WEs took part in the survey (mean age 51.6 years, SD 8.64). Almost half of them were 45–55 years old (*n* = 99, 45.6%) and had completed primary education (*n* = 99, 45.6%). Most were weavers (*n* = 113, 52.1%), had worked 1 to 10 years (*n* = 141, 65.3%), and were married (*n* = 163, 75.1%) (Table 1).

**Table 1.** Demographic profiles of women entrepreneurs with uncorrected presbyopia (*n* = 217).

Demographics	Number of women entrepreneurs with uncorrected presbyopia (%)
**Age group (years)**	
35–44	54 (24.9%)
45–55	99 (45.6%)
Older than 55	64 (29.5%)
Mean age (standard deviation)	51.6 (8.46)
**Level of education**	
No formal education	25 (11.5%)
Did not complete primary education	25 (11.5%)
Completed primary education	99 (45.6%)
Completed secondary education	66 (30.4%)
*missing data = 2	
**Type of engaged work**	
Weaving	113 (52.1%)
Tailoring and sewing	63 (29.0%)
Pottery	9 (4.1%)
Producing oil and making soaps	20 (9.2%)
Farming	12 (5.5%)
**Years working as an entrepreneur**	
Less than a year	1 (0.5%)
1 to 10 years	141 (65.3%)
>10 to 20 years	57 (26.4%)
>20 to 30 years	14 (6.5%)
>30 to 40 years	3 (1.4%)
*missing data = 1	
**Marital status**	
Single	2 (0.9%)
Married	163 (75.1%)
Widowed	30 (13.8%)
Separated/divorced	22 (10.1%)
**Number of children**	
1 to 4	61 (28.1%)
5 to 8	115 (53.0%)
More than 8	37 (17.1%)
*missing data = 4	
**Number of dependents**	
None	14 (6.5%)
1 to 4	119 (54.8%)
5 to 8	75 (34.6%)
More than 8	9 (4.1%)
**Access to a mobile phone**	
Yes	191 (88.0%)
No	26 (12.0%)
**With distance refractive error**	
Yes	40 (18.4%)
No	177 (81.6%)

#### Women entrepreneurs’ SWB score and self-rated work performance

WEs had a mean SWB score of 3.32 (SD 1.10) before correction and 5.99 (SD 1.13) shortly after correction, giving a significant improvement of 2.67 (95% CI 2.49–2.85, *p* < .001). WEs had a mean self-rated current work performance score of 4.62 (SD 1.36) before correction and 5.47 (SD 1.35) after correction, with a significant change of 0.85 (95%CI 0.66–1.04. *p* < .001) (Table 2).

**Table 2.** Subjective wellbeing and work performance before and after correction.

	Women entrepreneurs with uncorrected functional presbyopia (*N* = 217)	p-value*
**Mean subjective wellbeing score**		
(a) Before eyesight is corrected	3.32 ± 1.10	
(b) When corrected	5.99 ± 1.13	
**Mean difference in subjective wellbeing score**		
- Perceived immediate change in wellbeing when eyesight is corrected Ʃ(b) – (a)/N	2.67 ± 1.32 (2.49, 2.85)	<0.001
**Mean work performance score**		
(c) Before eyesight is corrected	4.62 ± 1.36	
(d) When corrected	5.47 ± 1.35	
**Mean difference in work performance score** - Perceived immediate change in self-rated work performance when eyesight is corrected Ʃ(c) – (d)/N	0.848 ± 1.41 (0.66, 1.04)	<0.001

*paired sample t-test was used.

Most WEs (*n* = 90, 87.6%) rated their baseline SWB score < 5, with no significant differences between the various demography characteristics and SWB scores. Over half of WEs (*n* = 121, 55.8%) gave self-rated current work performance scores < 5. Compared to those with 1 to 10 years of work experience, WEs with > 10 to 20 years and > 20 to 30 years of work experience were 3 times (95%CI 1.54–5.83) and 4.29 times (95%CI 1.15–16.0), respectively more likely to have low work performance scores (Table 3).

**Table 3.** Prevalence of, and factors associated with women entrepreneurs with functional presbyopia rated subjective wellbeing and work performance scores less than 5.

	Women entrepreneurs with a wellbeing score of < 5 * (*N* = 190)	Odds Ratio (95% CI)^	Women entrepreneurs with a work performance score of < 5 * (*N* = 121)	Odds Ratio (95% CI)^
**Age group (years)** 35–44 45–55 Older than 55	44 (23.2%) 91 (47.9%) 55 (28.9%) p = .156	Reference 2.59 (0.95, 7.01) 1.39 (0.52, 3.72)	25 (20.7%) 59 (48.8%) 37 (30.6%) p = .264	Reference 1.71 (0.88, 3.34) 1.59 (0.77, 3.30)
**Level of education** No formal education Did not complete primary education Completed primary education Completed secondary education	24 (12.8%) 22 (11.7%) 87 (46.3%) 55 (29.3%) p = .439	Reference 0.32 (0.03, 3.16) 0.30 (0.04, 2.44) 0.21 (0.03, 1.71)	17 (14.0%) 11 (9.10%) 59 (48.8%) 34 (28.1%) p = .264	Reference 0.37 (0.12, 1.17) 0.69 (0.27, 1.76) 0.50 (0.19, 1.32)
**Type of craftwork engaged** Weaving Sewing Pottery Producing oil and making soaps Farming	98 (51.6%) 56 (29.5%) 8 (4.20%) 16 (8.40%) 12 (6.30%) p = .567	Reference 1.22 (0.47, 3.18) 1.22 (0.14, 10.5) 0.61 (0.18, 2.08) N/A	66 (54.5%) 35 (28.9%) 4 (3.30%) 10 (8.30%) 6 (5.00%) p = .875	Reference 0.89 (0.48, 1.66) 0.57 (0.15, 2.24) 0.71 (0.28, 1.85) 0.71 (0.22, 2.35)
**Years working as women entrepreneurs** Less than a year 1 to 10 years >10 to 20 years >20 to 30 years >30 to 40 years	1 (0.50%) 124 (65.6%) 48 (25.4%) 13 (6.90%) 3 (1.60%) p = .822	N/A Reference 0.73 (0.31, 1.75) 1.78 (0.22, 14.5) N/A	1 (0.80%) 65 (53.7%) 41 (33.9%) 11 (9.10%) 3 (2.50%) **p = .002**	N/A Reference 3.00 (1.54, 5.83)^#^ 4.29 (1.15, 16.0) ^#^ N/A
**Marital status** Single Married Widowed Separated/divorced	2 (1.10%) 146 (76.8%) 24 (12.6%) 18 (9.50%) p = .372	N/A Reference 0.47 (0.17, 1.30) 0.52 (0.16, 1.73)	-93 (76.9%) 15 (12.4%) 13 (10.7%) p = .371	N/A Reference 0.75 (0.35, 1.64) 1.09 (0.44, 2.69)
**Number of children** 1 to 4 5 to 8 More than 8	51 (27.3%) 100 (53.5%) 36 (19.3%) p = .194	Reference 1.31 (0.55, 3.12) 7.06 (0.87, 57.6)	29 (24.4%) 67 (56.3%) 23 (19.3%) p = .452	Reference 1.54 (0.83, 2.88) 1.81 (0.79, 4.17)
**Number of dependents** None 1 to 4 5 to 8 More than 8	13 (6.80%) 104 (54.7%) 65 (34.2%) 8 (4.20%) p = .93	Reference 0.53 (0.07, 4.38) 0.50 (0.06, 4.25) 0.62 (0.03, 11.3)	10 (8.30%) 68 (56.2%) 39 (32.2%) 4 (3.30%) p = .497	Reference 0.53 (0.16, 1.80) 0.43 (0.13, 1.51) 0.32 (0.06, 1.85)
**Access to a mobile phone** Yes No	167 (87.9%) 23 (12.1%) p = .88	Reference 1.10 (0.31, 3.95)	105 (86.8%) 16 (13.2%) p = .527	Reference 1.31 (0.57, 3.04)

*Chi-square was used; ^ Multinomial regression was used; ^#^*p* <.005; N/A: odds ratio too small to report.

#### Impact of uncorrected presbyopia on women entrepreneurs’ daily lives

Around one in four WEs reported having severe difficulties seeing close objects (*n* = 60, 27.6%), severe difficulties in reading ordinary-size print (*n* = 54, 24.9%), and difficulties with sewing or using hand tools (*n* = 55, 25.3%). A third of WEs had severe difficulties reading mobile phone screens (*n* = 70, 32.2%) and often or very often asked for help from others due to poor vision (*n* = 73, 33.7%). About 73% (*n* = 156) reported being frustrated a lot of the time by their poor vision. Lastly, most WEs reported that their vision problems limited the type and volume of work they could do (*n* = 163, 85.2%), and the amount of time spent on their work, at least some of the time (*n* = 149, 68.6%) (Table 4).

**Table 4.** Self-rated effects of presbyopia on women entrepreneurs’ daily lives (*n* = 217).

Statements	None or mild difficulty N (%)	Moderate Difficulty N (%)	Severe difficulty or cannot do it at all N (%)
1. How much difficulty do you have in seeing close objects?	74 (34.1%)	83 (38.2%)	60 (27.6%)
2. Because of your eyesight, how much difficulty do you have in looking after your appearance?	104 (47.9%)	78 (35.9%)	35 (16.2%)
3. How much difficulty do you have reading ordinary-size print, such as a label on a food package?	95 (43.8%)	68 (31.1%)	54 (24.9%)
4. How much difficulty do you have in sewing or using hand tools?	72 (33.2%)	90 (41.5%)	55 (25.3%)
5. How much difficulty do you have reading the display on your mobile phone?	146 (66.3%)	1 (0.50%)	70 (32.2%)
**Statements**	**Never or rarely** **N (%)**	**Sometimes** **N (%)**	**Often or very often** **N (%)**
6. Because of your poor eyesight, how often do you need to ask for help from others in your daily activities?	62 (28.5%)	82 (37.8%)	73 (33.7%)
**Statements**	**Definitely or mostly true** **N (%)**	**Not Sure** **N (%)**	**Mostly or definitely false** **N (%)**
7. I feel frustrated a lot of the time because of my poor eyesight”.	156 (72.9%)	16 (7.4%)	45 (20.8%)
**Statements**	**None or a little of the time** **N (%)**	**Some of the time** **N (%)**	**Most or all of the time** **N (%)**
8. In the past 2 weeks, how often did vision problems limit the kind or amount of work you could do?	54 (24.9%)	113 (52.1%)	50 (23.1%)
9. In the past 2 weeks, have you been limited in how long you can work or do other activities because of your vision?	68 (33.3%)	109 (50.2%)	40 (18.4%)

#### Findings from semi-structured interviews

The age of WEs interviewed ranged from 40 to 63 years, with a mean age of 49. Nineteen WEs were between 40 and 55, while five were over 55. Two themes emerged including: economic implications of vision impairment and non-economic implications of vision impairment. Illustrative quotes are shown in Table 5.

#### Economic implications

Poor vision at a close distance reduced the efficiency and quality of the WEs’ craft production. Because of their poor vision at a close distance, they took longer to produce their crafts (Quote 1). For some, their poor vision at a close distance affected the quality of their work (Quote 2). The inability to produce quality crafts efficiently further reduced WEs’ income. The WEs repeatedly mentioned that poor vision had led to their inability to produce crafts that were good quality and further reduced their income (Quote 3) or they stopped working entirely (Quote 4). One WE specified how she had difficulties with marketing her business on social media because she could not see well (Quote 5).

#### Non-economic implications

- Women entrepreneurs experienced poor physical functionality and health (functional near and distance vision and symptomatic eye conditions), leading to a reduced ability to perform daily activities.
  Women entrepreneurs with poor near and distance vision faced challenges in their craft work (Quote 6), daily living (Quote 7), and mobility (Quote 8). In addition, WEs experienced vision-related physical symptoms such as experiencing headaches and tiredness (Quote 9) and had to stop working on their tasks (Quote 10).
- Poor vision at a close distance can cause poor psychological wellbeing Vision impairment caused negative emotions and reduced self-confidence. Some WEs felt that their inability to see or do certain things made them feel stressed, embarrassed, and worried about making mistakes (Quote 11). Consequently, their self-confidence declined due to reduced work performance and their inability to perform simple tasks (Quote 12).
- Poor vision caused WEs’ to be dependent on others for help Women entrepreneurs had to depend on others to help them with certain tasks to continue working (Quote 13). Sometimes the WEs’ reliance on others’ help strained their familial relations (Quote 14). Although the majority of WEs reported being dependent on others for work-related tasks, others mentioned needing additional assistance reading messages or loading an airtime voucher on their mobile phone (Quote 15).
- Poor vision at a close distance can bring about social and political challenges Because of vision impairment, attending social activities was a challenge but good vision would overcome this challenge (Quote 16). Interestingly, one craftswoman felt that vision impairment had affected her social and political work (Quote 17).

**Table 5.** Quote number, illustrative quotes and women entrepreneur’s descriptions from the qualitative semi-structured interviews.

Quote number	Illustrative quotes	Descriptions (Craft_Age in years_Location)
Quote 1	*“It affected me somehow because the work that has to be done in two days with someone who does not have an eye problem, it takes me three to four days to accomplish that work.”*	Potter_48_Unguja
Quote 2	*“I was not able to do my work properly, for example … when I want to stitch clothes.”*	Tailor_44_Unguja
Quote 3	*“I was not doing my work properly, so the income also (negatively) affected”*	Weaver_40_Unguja
Quote 4	*“when (poor vision) happens I cannot go on working.”*	Potter_45_Pemba.
Quote 5	*“ … my vision impairment hindered me to advertise my business on social media.”*	Weaver_41_Pemba
Quote 6	*“ … I was struggling in reading and putting the thread into the needle.”*	Tailor_44_Unguja
Quote 7	*“I can’t see the name of the place that was labelled in front of the car, so I don’t know whether that car is the right one to take…”*	Potter_45_Pemba
Quote 8	*“I had trouble walking because even shopping was a problem.”*	Tailor_46_Pemba
Quote 9	*“ … feeling headache and sometimes feeling very tired after forcing myself to continue with tailoring while eyes are already tired.”*	Tailor_40_Pemba
Quote 10	*“ … cannot go on working.”*	Potter_45_Pemba
Quote 11	*“because I was worried that my business would not run smoothly as a start for fear of making mistakes that would ruin my crafts with an eye problem … ”*	Tailor_46_Pemba
Quote 12	*“I could not see even a voucher to recharge my airtime, … that reduced my confidence”*	Weaver_40_Unguja
Quote 13	*“ … if there is no one to help me [to put] the thread, I could not proceed. I just quit working.”*	Weaver_58_Unguja
Quote 14	*“ … for example, even in pinpointing the sewing injection, it was very stressful for me, and I had to call the kids who sometimes refuse and run, it was bothering me.”*	Tailor_48_Pemba
Quote 15	*“if I want to read SMS, I had to ask my children for … help to read for me or putting voucher into my phone.”*	Weaver_40_Unguja
Quote 16	*“Yes, my involvement in social activities will increase, because I was avoiding some of the journeys because of my eyesight problem.”*	Potter_45_Pemba
Quote 17	*“ … I am an activist involved in social and political work with women, so in reading, I was not comfortable at all.”*	Weaver_58_Unguja

## Discussion

Our study aimed to further understand the relationship between presbyopia and its effects on worker productivity and SWB using a mixed-methods approach. We found that more than half of our cohort reported low SWB and work performance before correction (Cantril’s score < 5). A majority of WE’s then reported significant improvements in SWB and work performance scores post correction. Poor vision was found to negatively impact WE’s daily lives, reducing functionality, income, and psychological wellbeing while also creating new relational and social challenges.

No study in eye health has measured the impact of functional presbyopia on SWB. The closest comparisons were studies that measured wellbeing in terms of QoL,^6–8,13^ which showed that older people with presbyopia had reduced QoL and that presbyopia correction improved their QoL. Similarly, our findings showed that WEs believed their SWB could improve significantly after correction.

WEs perceived their SWB and work performance to be low prior to presbyopia correction. Those engaged in their work for a longer time were more likely to rate their work performance lower than their counterparts. We presume this could be because those who have engaged in their work longer were more likely to be older than their counterparts and would have higher presbyopia and thus affecting their near-work performance more significantly. Secondly, more experienced WEs might have higher work performance expectations than their less experienced counterparts.

Uncorrected functional presbyopia made craft-making more difficult for WE’s, reducing their work productivity and income. These issues are inter-related since these women struggled to do tasks required for their crafts, such as having problems seeing the thread, which slowed down their production time. Presbyopic WEs also took longer to make crafts because they often needed to wait for others to help them. Therefore, WEs sold fewer crafts which resulted in decreased income. Our findings of the economic implications of uncorrected presbyopia are supported by other studies in LMICs.^2–4^

Findings in this study correlate with those found in Bangladesh, where female garment workers with presbyopia earned $6.51 per month less than those without presbyopia after adjusting for possible confounders.^9^ The reduction in income amongst garment workers was associated with decreased productivity since workers were paid according to what they produced. Similarly, presbyopia caused a reduction in the quality and number of crafts made by WEs. Findings related to the economic consequences of uncorrected presbyopia in WEs are supported by other studies, comprising mainly female participants, in LMICs that looked at the effects of presbyopia on productivity and income.^10–12^

Presbyopia made business advertising on social media difficult for WEs since the fine print on a mobile phone could not be seen. As e-commerce in Africa continues to grow, with mobile e-commerce leading online trading between businesses and consumers,^23^ an inability to use mobile phones creates further barriers to economic development amongst women. This highlights how presbyopia can not only limit physical work activities but also impede WEs from expanding their businesses and generating more income.

Reduced income would have implications for WEs and their families. Firstly, many households in Zanzibar are headed by women over 44,^24^ so their dependents rely on their income for food and other household expenditures. Women in rural areas of Zanzibar are twice as likely to be basic needs poor,^25^ which is further exacerbated by women earning less money on average. Additionally, reduced earnings could keep households from escaping poverty or drive them into further poverty.

Reduced income would likely delay improvements in women’s economic empowerment and increase the gender gap. Zanzibar forms part of Tanzania, and since there is a lack of up-to-date gender statistics for Zanzibar,^25^ the Tanzanian Gender Inequality Index (GII) was used. The GII in Tanzania^26^ shows how the country fares worse than the world average on gender inequality. Further, there have not been improvements in reducing the gender gap in Tanzania for over a decade.^26^ This hinders progress in achieving SDG 5 (gender equality) by 2030.^27^

The physical and psychological wellbeing of WEs were negatively affected by presbyopia, contributing to reduced SWB. WE’s believed that presbyopia reduced their self-confidence and caused negative emotions of embarrassment, worry, and stress. These negative emotions often stemmed from an inability to perform the tasks they were supposed to be skilled in as well as their dependence on others for assistance.

### Limitations

Our study was limited to older women entrepreneurs mostly involved in craft trade. Future studies could assess women in other trades to compare findings across settings. This study only assessed immediate effects of the presbyopia correction on the SWB and work performance. We recommend SWB and work performance scores to be studied longitudinally to understand the effects of presbyopia correction with a larger-scale randomised trial (if possible) to validate the study findings. Due to unavailability, it resulted in a larger proportion of WEs between the ages of 40 and 55 than those over 55. Therefore, perceptions from younger WEs may have been unrepresented.

## Conclusion

This study demonstrated the immediate positive impact of correcting presbyopia on WE’s SWB and work performance scores. Uncorrected presbyopia has negatively affected economic, physical and psychosocial aspects of WEs’ lives. More research is needed to understand the benefits of correcting presbyopia in longer follow-up.

## Data Availability

The datasets generated and/or analysed during the current study are available in the Queen’s University Belfast PURE repository, [TBC].
